# A Word for Women

**Published:** 1875-10

**Authors:** 


### A WORD FOR WOMAN.

If any lady leaves the Fair without examining the new DARNING-MACHINE, we advise her to take the earliest opportunity to repeat the visit, if for no other purpose than to witness the operation of this very useful instrument. The young lady who operates it—Miss Sadie Booth—makes her nimble fingers fly so fast that one can hardly tell how the work is done; but it is plain to see that a hole of large size, in a stocking, or in any garment, can be effectually darned in one minute or less. Every family should have one of these useful machines, as it will save much labor and patience and eye-sight. A child can work it, and its operation is very fascinating. Mrs. H. S. Hutchinson, No. 16 West 14th Street, New York, has these useful machines for sale. The price is $10.

